# Health-related quality of life measured with K-BILD is associated with survival in patients with idiopathic pulmonary fibrosis

**DOI:** 10.1186/s12890-024-03303-3

**Published:** 2024-09-30

**Authors:** Tuuli Rautajoki, Heidi A. Rantala, Eva Sutinen, Tiina Saarto, Kaisa Rajala, Ida Pesonen, Maria Hollmen, Marjukka Myllärniemi, Juho T. Lehto

**Affiliations:** 1 Individualized Drug Therapy Research Program, Faculty of Medicine, University of Helsinki, Helsinki, Finland; 2 Department of Pulmonary Medicine, Heart and Lung Center, Helsinki University Hospital, Helsinki, Finland; 3 Department of Respiratory Medicine, Tampere University Hospital, Tampere, Finland; 4 Faculty of Medicine and Health Technology, University of Tampere, Tampere, Finland; 5 University of Helsinki, Helsinki, Finland; 6 Palliative Care Center, HUS Comprehensive Cancer Centre, Helsinki, Finland; 7 Wellbeing Services of Vantaa and Kerava, Vantaa, Finland; 8 Respiratory Medicine Unit, Department of Medicine Solna, Karolinska Institutet, Stockholm, Sweden; 9 Department of Respiratory Medicine and Allergy, Karolinska University Hospital, Stockholm, Sweden; 10 Palliative Care Centre, Tampere University Hospital, Tampere, Finland

**Keywords:** Idiopathic pulmonary fibrosis, Interstitial lung disease, Survival, Health-related quality of life, K-BILD, mMRC

## Abstract

**Background:**

Health-related quality of life (HRQoL) assessments and estimates of prognosis are needed for comprehensive care and planning of subsequent treatment in patients with idiopathic pulmonary fibrosis (IPF). We investigated HRQoL and its association with survival using a disease-specific tool in patients with IPF.

**Methods:**

The patients were recruited from the real-life FinnishIPF study in 2015. HRQoL was assessed with the King’s Brief Interstitial Lung Disease (K-BILD) questionnaire every six months for 2.5 years. Dyspnoea was assessed with the modified Medical Research Council (mMRC) dyspnoea scale. Survival was registered until 31 December 2022. Patient survival according to the K-BILD total score was evaluated using the Kaplan‒Meier method. The Friedman test was used to compare the K-BILD total scores longitudinally, and the Mann‒Whitney U test was used to compare the mMRC groups. *P* values < 0.05 were considered statistically significant.

**Results:**

The median K-BILD total score (*n* = 245) was 51.6. At baseline, patients in the highest HRQoL quartile (K-BILD scores 58.9–100) had a longer median survival time (5.3 years) than did those with scores of 51.7–58.8 (3.1 years), 45.7–51.6 (2.3 years), and 0.0–45.6 (1.8 years). A decrease in the K-BILD total score of ≥ 5 units in the preceding 12 or 24 months showed a trend towards poorer survival, although statistical significance was not reached. Ninety-four patients survived more than 2.5 years and had available K-BILD data at all time points. The K-BILD total score remained higher in patients with a baseline mMRC of 0–1 than in those with a mMRC of 2–4, and the total score decreased only modestly in both groups (median of 3.3 and 4.8 units in patients with mMRC scores of 0–1 and 2–4, respectively).

**Conclusions:**

In IPF, a reduced HRQoL is associated with impaired survival. A K-BILD total score less than approximately 50 units is associated with a median survival of approximately two years. In addition to assessing the treatment needs of patients with IPF using K-BILD, a decreased score may be useful for facilitating advance care planning and transplantation assessment.

**Supplementary Information:**

The online version contains supplementary material available at 10.1186/s12890-024-03303-3.

## Introduction

Idiopathic pulmonary fibrosis (IPF) is a chronic fibrosing interstitial lung disease (ILD) that, despite recent research emphasis, remains incurable [1–4]. The median survival time was less than five years in the Finnish IPF patient registry [5], which is comparable with that noted in other studies [1, 3, 6]. The available pharmacological antifibrotic therapies reduce the rate of decline in lung function, as estimated by the forced vital capacity (FVC) [7, 8]. In addition, it has been acknowledged that treating comorbidities is essential in IPF and can influence patient survival [9, 10]. Only some patients with IPF are referred to lung transplantation after careful selection [1, 11]. To identify novel approaches to the care of patients with IPF, more research attention is required to assess the health-related quality of life (HRQoL) of patients along the disease continuum [12] and to investigate whether HRQoL is associated with patient survival. Using HRQoL tools, personalized advance care planning (ACP) and palliative care can be developed to provide individualized treatment for patients’ symptoms and to preserve HRQoL.

As previously shown, HRQoL in patients with IPF is multidimensionally impaired compared to that in the general population, and the major symptom of IPF, dyspnoea, further impairs HRQoL [13]. However, patients with IPF seem to maintain their cognitive status [13], which allows them to have an active role in treatment discussions and can offer essential insights from a patient’s perspective to guide personalized medical care. Preserved cognition also allows patients with IPF to thoroughly complete HRQoL inquiries, which can be utilized as quick follow-up tools in the clinical setting. In IPF, the commonly used HRQoL tools have been either generic or a generic form of a disease-specific tool that was originally invented for obstructive pulmonary disease [14]. However, the King’s Brief Interstitial Lung Disease (K-BILD) questionnaire is a more recently validated self-assessed inquiry designed to specifically evaluate the HRQoL in patients with ILD [15]. Hence, this disease-specific inquiry needs assessment to test its suitability for broader clinical use.

In this study, we aimed to evaluate the HRQoL of patients with IPF using the ILD-specific K-BILD questionnaire and to assess whether HRQoL status at baseline or changes during two years in HRQoL are associated with survival in patients with IPF. The results might be useful for physicians taking care of patients with IPF in the comprehensive evaluation of HRQoL and the determination of optimal time points for ACP and, in some cases, for lung transplantation.

## Materials and methods

**Fig. 1:**
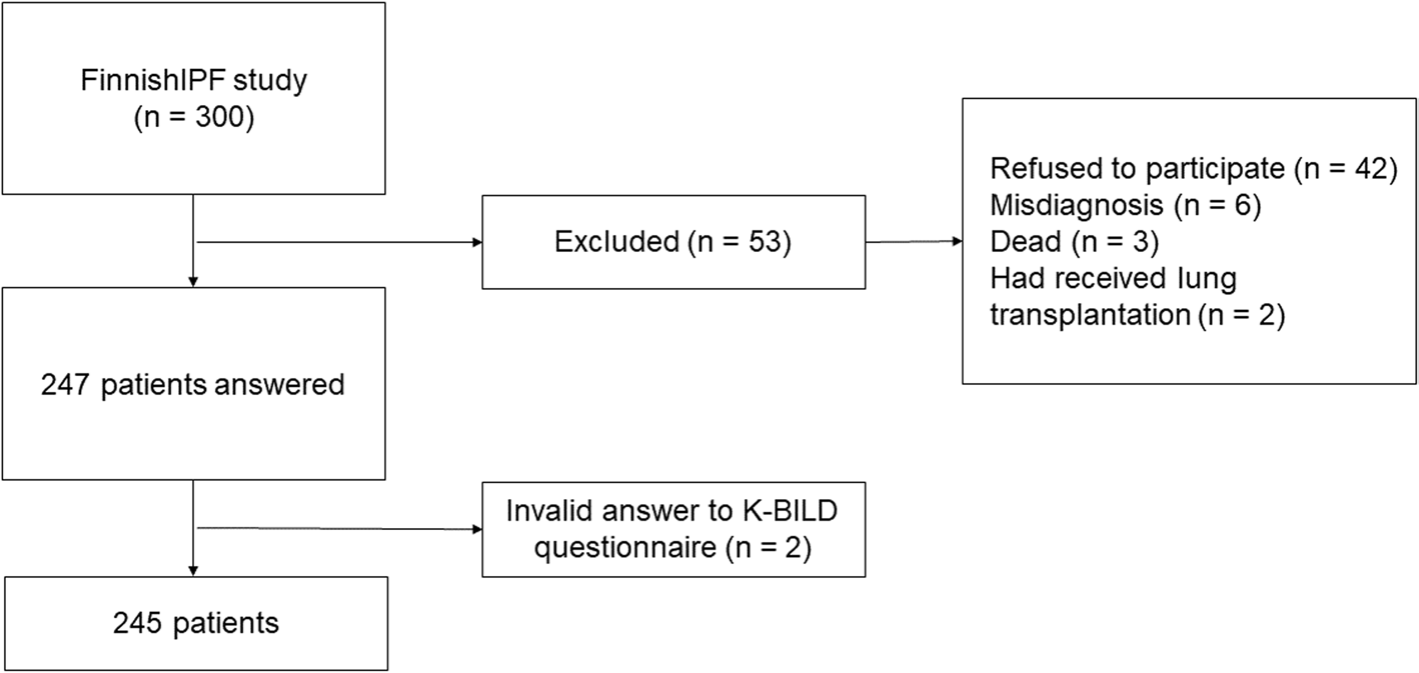
Flow chart of patient recruitment. Legend: K-BILD, King’s Brief Interstitial Lung Disease questionnaire

The FinnishIPF study originated in 2012 in which Finnish IPF patients’ sociodemographic and medical follow-up data have been collected to an electronic database [16]. The inclusion and exclusion criteria of the FinnishIPF have previously been presented in detail by Kaunisto et al. [5]. All IPF patients included in the study were identified according to the ATS/ERS 2011 criteria [1]. This quality of life (QoL) IPF study was initiated in April 2015, when the FinnishIPF had data on 300 patients [17]. These patients were contacted. Ultimately, 247 patients with IPF signed the informed consent form and composed the final QoL-substudy cohort [17]. The patient recruitment process is shown in Fig. 1.

Since 2015, the study participants have been asked to complete a sociodemographic study form and separate QoL questionnaires and symptom assessment tools (K-BILD, mMRC, Edmonton Symptom Assessment System (ESAS), RAND 36-Item Short Form Health Survey Questionnaire, and 15-dimensional instrument of health-related quality of life (15D)) twice a year. This study evaluates the K-BILD and the mMRC, while the results and details of the other questionnaires have been reported previously [13, 18, 19]. For survival analyses, patients were followed up until death, lung transplantation, or 31 December 2022.

### Study questionnaires

In this study, we report the results of the K-BILD questionnaire, which is a validated tool for specifically measuring the HRQoL status of patients with ILD [15]. The questionnaire consists of 15 items and three domains (breathlessness and activities, psychological, and chest symptoms) [15]. Both the total and domain scores of the K-BILD questionnaire range from 0 to 100 (lower scores indicate poorer health status) [15]. The minimal clinically important difference (MCID) in the K-BILD total score is five units [20]. To evaluate the longitudinal change in HRQoL with the K-BILD questionnaire, we included the K-BILD results until 31 October 2017 (i.e., a total interval of 30 months). The K-BILD has been translated into Finnish according to the multistage procedure instructed by the copyright holder (Additional file 1).

The mMRC is a self-assessed symptom-specific inquiry to estimate exertional dyspnoea [21, 22]. The mMRC scale ranges from 0 to 4. On this scale, 0 indicates “I only get breathless with strenuous exercise”; 1 indicates “I get short of breath when hurrying on the level ground or walking up a slight hill”; 2 indicates “I walk slower than people of the same age on the level ground because of breathlessness or have to stop for breath when walking at my own pace on the level”; 3 indicates “I stop for breath after walking about 100 m or after a few minutes on the level ground”; and 4 indicates “I am too breathless to leave the house or I am breathless when dressing or undressing” [21, 22].

### Statistical methods

The descriptive statistics of patient data are expressed as the numbers of observations with percentages, medians with interquartile ranges (IQRs), and means with standard deviations (SDs). The Shapiro‒Wilk test and visual estimations were performed to estimate the normal distribution of the variables. As most of the variables were non-normally distributed, mainly the non-parametric tests were used. The patients were divided into four groups, A, B, C, and D, according to the K-BILD total score median and IQR values. The Kruskal‒Wallis and Mann–Whitney U tests were used to compare the continuous variables, whereas Pearson Chi-Square test was used to compare the categorical variables. The Kaplan‒Meier method with a log-rank test was used to estimate the survival of the patients in the groups. A receiver operating characteristic curve (ROC) analysis was performed to estimate the capacity of the K-BILD total score to predict the survival time of less than two years. The optimal cut-off level was defined by the largest sum of sensitivity and specificity. In the longitudinal analyses of the survivors, the patients were divided into two groups according to their mMRC at baseline. The non-parametric Friedman test was used to compare the K-BILD total scores between the longitudinal time points. To comprehend the overall comparisons, pairwise comparisons were conducted, and *p*-values were Bonferroni adjusted. Furthermore, the Mann‒Whitney U test was used to compare the mMRC groups at each time point, and a Bonferroni correction was applied to the *p*-values. *P*-values < 0.05 were considered statistically significant. IBM SPSS Statistics for Macintosh (IBM Corp., Armonk, NY, USA), versions 25.0 and 29.0.0.0, was used for the statistical analyses.

### Ethical considerations

The Finnish National Institute for Health and Welfare (Finnish acronym, THL) has approved patient record screening in hospitals to identify patients for the FinnishIPF study (Dnro THL/568/5.05.00/2020 18.3.2020). The medical ethical committee of the Helsinki University Hospital approved this QoL research (Dnro HUS 381/13/03/01/14 28.1.2015). All included patients provided written informed consent for participation.

## Results

The questionnaires were sent to 300 patients with IPF, 42 of whom refused to participate, and 11 of whom were excluded. Thus, 245 patients were included in this study (Fig. 1). The characteristics of the patients at baseline are shown in Table 1. Most of the patients had comorbidities, as only 19.2% (*n* = 47) of the patients had no diseases other than IPF. The median time from IPF diagnosis to study entry was 3.4 years (IQR 2.1–5.1). The patients’ median K-BILD total scores at baseline according to their mMRC were 58.1 (IQR 52.7–65.6, *n* = 110) for mMRC 0–1 and 46.9 (IQR 40.7–51.2, *n* = 122) for mMRC 2–4. The response to the mMRC inquiry was missing at baseline in 13 patients.

**Table 1:** Patient characteristics at baseline

Total (n)	245
Gender, n (%)	
Females	83 (33.9)
Males	162 (66.1)
Age, Mean (SD), years	74.3 (8.7)
K-BILD, Median (IQR)	
Total score	51.6 (45.6–58.8)
Psychological	49.1 (43.8–60.8)
Breathlessness and activities	37.8 (22.9–48.0)
Chest symptoms	63.7 (44.0–73.4)
mMRC, n (%)^a^	
0	28 (11.4)
1	82 (33.5)
2	71 (29.0)
3	34 (13.9)
4	17 (6.9)
Smoking status, n (%)^b^	
Never	116 (47.3)
Former	105 (42.9)
Current smoker	24 (9.8)
Comorbidities, n (%)	
Hypertension	101 (41.2)
Coronary artery disease	54 (22.0)
Diabetes mellitus	48 (19.6)
COPD	43 (17.6)
Asthma	23 (9.4)
Cancer	41 (16.7)
Lung function^b^	
FVC Median (IQR) % of predicted^c^	81.0 (70.0–94.0)
DLCO Median (IQR) % of predicted^d^	58.0 (50.0–70.0)

*SD* Standard deviation, *K-BILD* King’s Brief Interstitial Lung Disease questionnaire, *IQR* Interquartile range, *mMRC* Modified Medical Research Council dyspnea scale, *COPD* Chronic obstructive pulmonary disease, *FVC* Forced vital capacity, *DLCO* Diffusing capacity of the lungs for carbon monoxide, *IPF* Idiopathic pulmonary fibrosis

^a^Data missing for 13 patients (5.3%)

^b^Documented simultaneously with IPF diagnosis

^c^Data missing for 16 patients (6.5%)

^d^Data missing for 35 patients (14.3%)

### Patient survival

Of the 245 patients, 78.0% (*n* = 191) had died, and 3.7% (*n* = 9) had received lung transplantation by the end of the follow-up (i.e., at the end of 2022). The median survival time was 2.9 years (IQR 1.6–6.1). The median survival time was longer (*p* < 0.001) in patients with a baseline mMRC of 0–1 (4.8 years (IQR 2.6-NA)) than in patients with a baseline mMRC of 2–4 (2.0 years (1.0–3.9)). The median ages were 73.2, 78.2, 73.6, and 75.7 years in patients included in Groups A (K-BILD total scores 58.9–100.0), B (K-BILD total scores 51.7–58.8), C (K-BILD total scores 45.7–51.6), and D (K-BILD total scores 0.0–45.6), respectively (*p* = 0.17 for the difference between groups). The detailed patient characteristics according to the K-BILD groups is shown in Table 2. Significant differences were observed in pulmonary function (FVC% of predicted, *p* = 0.005 and DLCO% of predicted, *p* < 0.001) and in the prevalence of coronary artery disease (*p* = 0.027).

**Table 2:** Baseline characteristics of the patients according to the K-BILD groups

	Group A	Group B	Group C	Group D	*P*-value
Total (n)	57	62	65	61	
Gender, n (%)					0.515
Females	17 (29.8)	18 (29.0)	26 (40.0)	22 (36.1)	
Males	40 (70.2)	44 (71.0)	39 (60.0)	39 (63.9)	
Age, Mean (SD), years	73.5 (8.4)	76.3 (9.5)	73.5 (8.5)	73.8 (8.3)	0.167
Smoking status, n (%)					0.877
Never	28 (49.1)	31 (50.0)	27 (41.5)	30 (49.2)	
Former	22 (38.6)	27 (43.5)	31 (47.7)	25 (41.0)	
Current smoker	7 (12.3)	4 (6.5)	7 (10.8)	6 (9.8)	
Comorbidities, n (%)					
Hypertension	27 (47.4)	20 (32.3)	28 (43.1)	26 (42.6)	0.378
Coronary artery disease	6 (10.5)	11 (17.7)	18 (27.7)	19 (31.1)	0.027
Diabetes mellitus	6 (10.5)	14 (22.6)	15 (23.1)	13 (21.3)	0.268
COPD	5 (8.8)	10 (16.1)	15 (23.1)	13 (21.3)	0.165
Asthma	2 (3.5)	4 (6.5)	10 (15.4)	7 (11.5)	0.111
Cancer	8 (14.0)	12 (19.4)	9 (13.8)	12 (19.7)	0.712
Lung function					
FVC Median (IQR) % of predicted	88.0 (76.5–99.5)	81.0 (72.0–94.5)	80.0 (68.5–90.5)	74.5 (65.8–88.5)	0.005
DLCO Median (IQR) % of predicted	63.0 (54.5–77.5)	58.5 (51.0–72.5)	59.0 (50.0–68.3)	52.0 (43.5–59.5)	< 0.001

*COPD* Chronic obstructive pulmonary disease, *FVC* Forced vital capacity, *DLCO* Diffusing capacity of the lungs for carbon monoxide

**Fig. 2:**
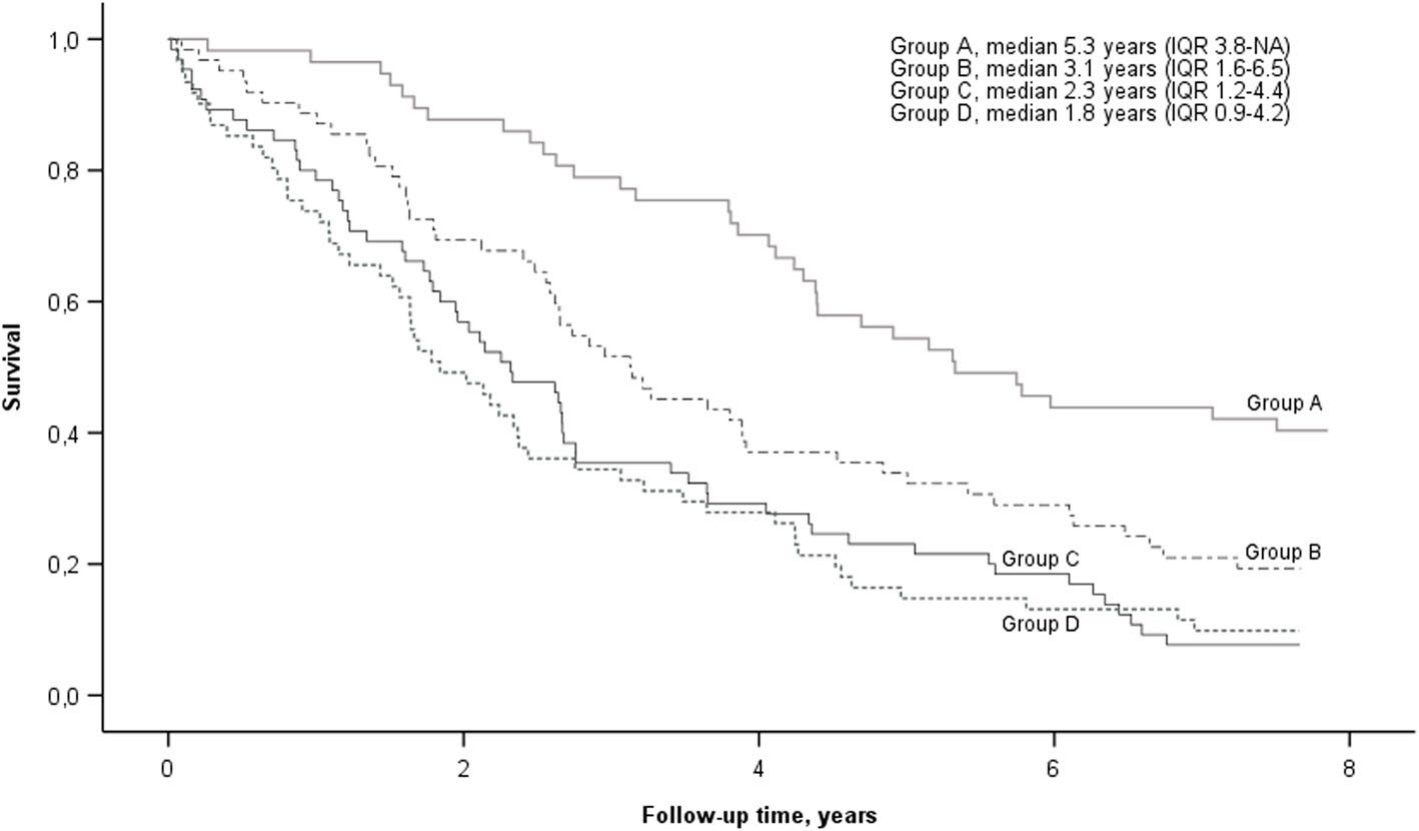
Kaplan–Meier survival curves of the total patient population according to their K-BILD total score. Legend: Group A had a K-BILD total score of 58.9–100 (*n* = 57), Group B had a K-BILD total score of 51.7–58.8 (*n* = 62), Group C had a K-BILD total score of 45.7–51.6 (*n* = 65), and Group D had a K-BILD total score of 0–45.6 (*n* = 61). Group A differed significantly from the other groups (A vs. B, *p* = 0.003; A vs. C, *p* < 0.001; and A vs. D, *p* < 0.001). The remainder of the pairwise comparisons were not statistically significant. All *p*-values can be found in Additional file 2. K-BILD, King’s Brief Interstitial Lung Disease questionnaire

The survival curves of the patients according to their baseline K-BILD total scores are illustrated in Fig. 2. The survival distributions of the four groups were statistically significantly different (χ^2^(3) = 33.888, *p* < 0.001). According to pairwise log rank comparisons with Bonferroni corrections, the survival of patients in Group A was statistically significantly longer than that of patients in the other groups (Groups A vs. B, *p* = 0.003; Groups A vs. C, *p* < 0.001; and Groups A vs. D, *p* < 0.001). Detailed information about the pairwise comparison analyses is shown in Additional file 2. A ROC curve analysis was performed to evaluate the capacity of the K-BILD total score to assess the survival of less than two years. (Additional file 3.) The area under the curve (AUC) was 0.689 (CI 0.621–0.757, *p* < 0.001). Estimated cut-off point with a maximum sum of sensitivity and specificity was a total score of 48 points (sensitivity 0.76 and specificity 0.58). The characteristics of the patients whose survival was less or more than two years are shown in Additional file 4.

**Fig. 3:**
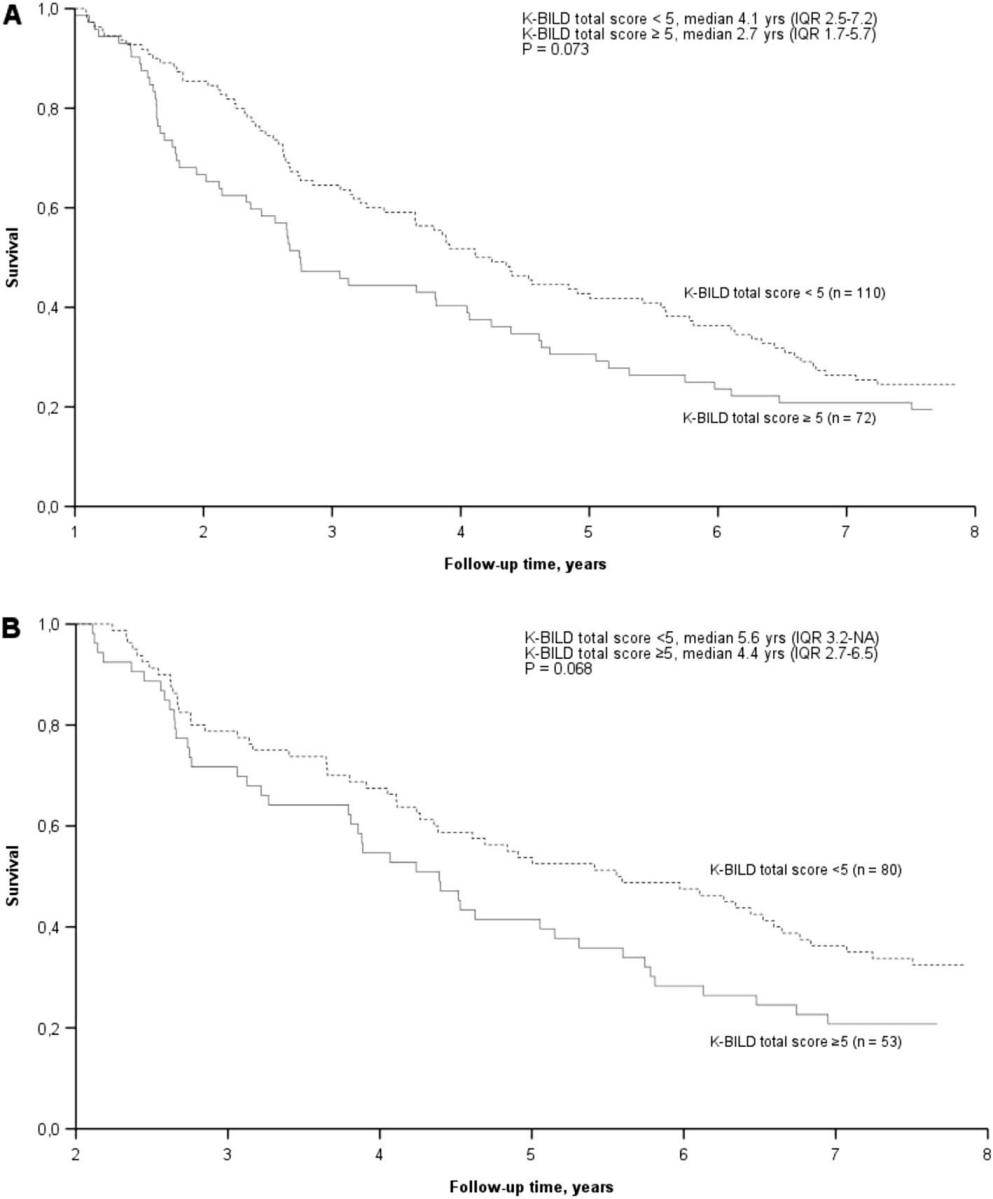
Survival according to a decrease in the K-BILD total score. Legend: Decrease in the K-BILD total score of more or less than 5 units during the previous A. 12 months and B. 24 months. K-BILD, King’s Brief Interstitial Lung Disease questionnaire

Figure 3 shows the survival curves of the patients with a decrease in the K-BILD total score of less than five and a minimum of five units (MCID) during the previous year and two years. After the initiation of the study, 39 patients died or received lung transplantation during the first year, and 46 died or received lung transplantation during the second year. A decreasing K-BILD score with a threshold of five showed a trend towards a lower survival, although the difference did not reach statistical significance.

### Longitudinal changes in K-BILD total scores in survivors

**Fig. 4:**
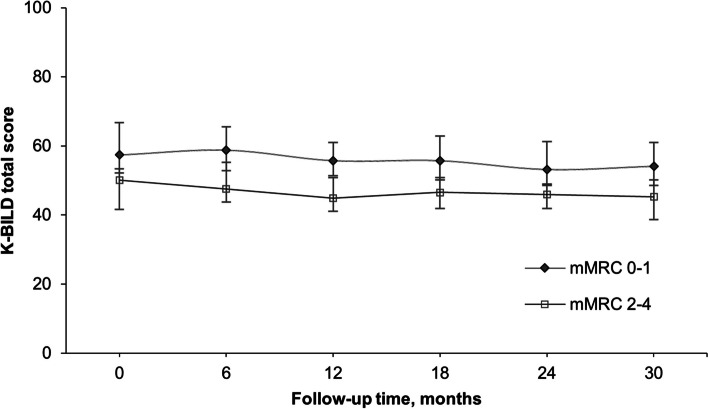
Longitudinal changes in K-BILD total scores (median, interquartile range (IQR)). Legend: The analyses included the patients who survived for 2.5 years and completed the questionnaire at each time point (*n* = 94). Patients who died were excluded. K-BILD, King’s Brief Interstitial Lung Disease questionnaire; mMRC, modified Medical Research Council dyspnoea scale

Of the 142 patients who survived until the end of October 2017, 94 patients (66.2%) had a K-BILD total score available at each of the six time points (0, 6, 12, 18, 24, and 30 months) and an mMRC score at baseline. We categorized the patients into two groups according to their baseline mMRC scores: mMRC 0–1 (*n* = 62, 66.0%) and mMRC 2–4 (*n* = 32, 34.0%). The longitudinal results of the K-BILD total scores are shown in Fig. 4. The median change in the K-BILD total score was -3.3 points in the mMRC 0–1 group and -4.8 points in the mMRC 2–4 group. The K-BILD total score decreased statistically significantly during the 2.5-year interval in both mMRC categories (mMRC 0–1, χ^2^(5) = 56.519, *p* < 0.001; mMRC 2–4, χ^2^(5) = 23.253, *p* < 0.001). The post hoc analysis of the differences in the K-BILD total scores between the different time points is shown in Additional file 5. The K-BILD total scores in the mMRC 0–1 and mMRC 2–4 groups differed significantly at each of the six time points (all *p*-values < 0.001).

## Discussion

Our results from the Finnish IPF patient cohort reinforce the previous findings of impaired HRQoL in patients with IPF [17, 23, 24]. We showed that a deteriorated HRQoL measured with the ILD-specific K-BILD is associated with shorter survival. In addition, the longitudinal decrease in the K-BILD total score during the follow-up was associated with a trend towards shorter survival. Our results also indicate that there seems to be a group of patients with IPF who maintain a rather steady HRQoL. Our study emphasizes the value of measuring HRQoL using K-BILD in patients with IPF not only to evaluate the need for comprehensive care but also to recognize poor survival.

In our earlier study, we used the generic HRQoL instrument 15D and showed that the HRQoL of Finnish IPF patients was multidimensionally impaired compared with that of the age- and gender-standardized population [13]. Our current study allows us to compare our Finnish IPF patients’ K-BILD results (median (IQR): total 51.6 (45.6–58.8), psychological 49.1 (43.8–60.8), breathlessness and activities 37.8 (22.9–48.0), and chest symptoms 63.7 (44.0–73.4)) with those of previous studies. In a Greek IPF study, the K-BILD total score was high (mean (SD), 69.3 (18.7); separate domain scores were not mentioned) [25]. In that study, enrolment occurred during the first clinic visit [25]. However, our K-BILD baseline scores were not recorded at the time of diagnosis, which at least partly explains the lower K-BILD score in our cohort. Additionally, the K-BILD scores in a relatively small Asian multiracial IPF patient cohort (*n* = 27) were also greater than those in our patient cohort (K-BILD scores, mean (SD): total 61.7 (14.1), breathlessness and activities 50.5 (16.3), psychological 63.5 (18.7), and chest symptoms 76.2 (21.6)) [23]. In that study, patients who did not have pulmonary function tests or available HRQoL data within six months were excluded [23]. The lung function of these Asian IPF patients (mean (SD) % of predicted, FVC 78.1 (20.5) and DLCO 50.7 (14.4)), as well as the lung function of the Greek patients (mean (SD) % of predicted, FVC 77.0 (21.2) and DLCO 51.7 (19.4)), did not differ markedly from the levels recorded at the time of IPF diagnosis in our study [23, 25]. In these two studies, the patients were in an early phase of the IPF disease trajectory, whereas our baseline results represent a real-life unselected population of patients with IPF [23, 25]. In our study, the time interval from the IPF diagnoses to the first measurement of K-BILD (baseline) varied between the participants. Therefore, IPF had probably progressed, and the lung function decreased by the time of the baseline K-BILD measurement in some of our patients. This probably explains the relatively low K-BILD scores compared to the studies mentioned above. In a Danish study of 150 patients with IPF, the mean K-BILD total score was 58.3 (SD 12.4) [26]. Additionally, in that study, the median time from diagnosis to study entry was shorter than that in our study (0.5 vs. 3.4 years) [26]. However, the combined K-BILD results of French, Italian, Dutch, and Swedish IPF patients (K-BILD mean, (SD): total 51.9 (22.2), breathlessness and activities 36.5 (28.5), psychological 52.2 (24.7), chest symptoms 66.5 (23.9)) were more parallel to our K-BILD scores [27]. In summary, our K-BILD results were similar to those of earlier studies considering the different time points used for the HRQoL assessment. In our study and previous studies, breathlessness and activities was the most severely impaired K-BILD domain, followed by the psychological domain [23, 27]. This highlights the need for psychosocial support in addition to the management of respiratory symptoms and interventions aimed at maintaining physical function in patients with IPF.

In this study, we showed the association between impaired HRQoL measured with K-BILD and reduced survival in patients with IPF. Previously, Kim et al. have examined the properties of some patient-reported outcome measures in IPF [28]. In their mortality analysis with K-BILD, they reported that patients with IPF who were in the poorest health tertile had lower survival than patients in the best tertile [28]. The survival results in that study are consistent with our study. However, we had a remarkably longer follow-up time (1.0 vs. 7.5 years), and we compared four groups with different K-BILD total score levels instead of three, which reinforces the significance of our study [28]. In their study, Prior et al. also suggested an association between K-BILD and mortality; however, in further analyses, this association did not reach statistical significance [26]. This study included a smaller patient cohort (*n* = 124) and a shorter follow-up time (one year) than our study [26]. To our knowledge, only those two studies and our present study have investigated the association of K-BILD with the survival of patients with IPF [26, 28]. In contrast, some studies have evaluated the association of the scores achieved on the non-ILD-specific HRQoL instrument, the St. George’s Respiratory Questionnaire (SGRQ), with survival [29–31]. For instance, a retrospective study by Furukawa et al. indicated that the total SGRQ is a predictor of mortality in IPF patients, and, in particular, a score greater than 30 was associated with a shorter survival time [29]. A German longitudinal IPF registry study showed that greater deterioration in the QoL at the last death preceding follow-up was associated with a greater risk of mortality [30]. In contrast to these results, in detailed analyses of the Australian IPF registry study, an association between SGRQ total level at baseline and mortality risk was not clearly revealed [31]. Our results with long-term follow-up and a relatively large number of patients strongly emphasize the greater mortality risk of patients with IPF with worse HRQoL measured using the ILD-specific K-BILD tool. We recognise that our ROC curve analysis showed only modest sensitivity and specificity values to predict survival of less than two years with the K-BILD total score cut-off of 48. However, we still tend to suggest that when the K-BILD total score is less than approximately 50, patients with IPF have a marked risk of dying within approximately two years. Therefore, listing for transplantation and starting a discussion on advance care planning might be reasonable at this time point. Nevertheless, more research is needed to find novel approaches to estimate the remaining lifetime in IPF.

We complemented our survival data by studying the association of the change in K-BILD total score over time with survival with a threshold of five (MCID) [20]. Recently, Birring et al. showed that in progressive fibrosing interstitial lung diseases, the meaningful change in the K-BILD total score is ≥ –2 [32]. However, patients with IPF were not included in those analyses [32]; hence, we decided to use the previously reported MCID value of five [20] in our study. Twelve and 24 months were chosen as the time points for examining the impact of decreasing K-BILD total scores on patient survival in the early phase of IPF. Additionally, the study drop-outs (mainly due to mortality) during the latter follow-up would have further limited the statistical value of the results. In both analyses, a clear trend was observed indicating that deterioration in HRQoL seems to be associated with impaired survival despite the baseline HRQoL. Both analyses nearly reached statistical significance. To our knowledge, our study is the first to report a possible association between reduced K-BILD total scores and reduced IPF survival. Hence, this should be noted as a novel and interesting finding that requires further research to be statistically validated.

Our longitudinal results indicate that there seems to be a subpopulation of patients with IPF with relatively long survival and rather well-preserved HRQoL. Our 2.5-year follow-up with long-term IPF survivors revealed that even though a statistically significant decrease in the HRQoL was observed in both mMRC categories, the change in the K-BILD total score did not reach the MCID level in either category. Although many patients with IPF suffer from impaired HRQoL and high symptom burden due to severe IPF, others may maintain somewhat satisfying lives for years. This may be referred to as a positive signal to patients and a source of hope at the time of diagnosis and during follow-up visits thereafter.

Dyspnoea is a significant symptom of IPF [1, 13]. Previously, using the generic HRQoL tool 15D, we showed that increased exertional dyspnoea (estimated as an mMRC ranging from 2 to 4) significantly impaired HRQoL, both at baseline and longitudinally [13]. In line with this study, we observed a similar effect with the ILD-specific tool K-BILD. An increased level of dyspnoea (mMRC 2–4) significantly worsened HRQoL, and this impact was observed until the end of the 2.5-year follow-up. To reduce the symptom burden and to maintain the HRQoL of patients with IPF through timely palliative care, patients’ subjective estimates of dyspnoea need constant observation.

The strength of our study is the use of a large cohort of patients with IPF in a prospective real-life nationwide research setting. In addition, our survival analyses had a remarkably long follow-up, i.e., seven and a half years, and the patients exhibited longitudinal commitment to research participation. Furthermore, a considerable strength is that the participants answered the study questionnaires at specific time points twice a year, which is seldom the case in real-life cohorts. However, a minor drop-out rate was a limitation of the study. More patients, especially in the longitudinal survival analyses, would probably have increased the statistical power and revealed the statistical significance of some of our results. Regardless, the results still show a recognizable trend between deteriorating HRQoL and worse survival, which provides a good investigational basis for further studies. Additionally, and most importantly, we were able to show that the survival of the patients with IPF worsened in relation to poorer HRQoL measured with the K-BILD. This finding strongly reinforces the relevance of HRQoL assessment in clinical practice; it can assist in showing the proper moment to proceed to major clinical decisions, such as ACP and, in some cases, being placed on the list for pulmonary transplantation.

## Conclusion

In IPF, a deteriorated HRQoL, measured with the ILD-specific tool K-BILD, is associated with impaired survival. A longitudinal decrease in the K-BILD total score might also reflect worse survival, but long-term survivors with quite stable HRQoL were also observed. The HRQoL assessment with K-BILD is recommended during the follow-up of patients with IPF to determine the need for supportive care and to recognize decreasing survival. A low K-BILD total score of 50 might represent a suitable threshold for ACP discussions and listing for lung transplantation, if appropriate.

## Supplementary Information

Supplementary Material 1.

Supplementary Material 2.

Supplementary Material 3.

Supplementary Material 4.

Supplementary Material 5.

## Data Availability

The datasets generated and analysed during the current study are not publicly available. Finland has a small population and idiopathic pulmonary fibrosis is a rare disease. By sharing data, the privacy of the study participants could be compromised.

## Abbreviations

HRQoL: Health-related quality of life
IPF: Idiopathic pulmonary fibrosis
FinnishIPF: The FinnishIPF study
K-BILD: King’s Brief Interstitial Lung Disease questionnaire
mMRC: Modified Medical Research Council dyspnoea scale
IQR: Interquartile range
ILD: Interstitial lung disease
FVC: Forced vital capacity
ACP: Advance care planning
QoL: Quality of life
ESAS: Edmonton Symptom Assessment System
15D: 15-Dimensional instrument for health-related quality of life
MCID: Minimal clinically important difference
SD: Standard deviation
ROC: Receiver operating characteristic curve
THL: Finnish acronym for The Finnish National Institute for Health and Welfare
HUS: Helsinki University Hospital
NA: Not applicable
AUC: Area under the curve
SGRQ: St. George’s Respiratory Questionnaire
COPD: Chronic obstructive pulmonary disease
DLCO: Diffusing capacity of the lungs for carbon monoxide

## References

[1] Raghu G, Collard HR, Egan JJ, Martinez FJ, Behr J, Brown KK, et al. An Official ATS/ERS/JRS/ALAT statement: idiopathic pulmonary fibrosis: evidence-based guidelines for diagnosis and management. Am J Respir Crit Care Med. 2011;183(6):788–824. 10.1164/rccm.2009-040GL. 21471066 10.1164/rccm.2009-040GL PMC5450933

[2] Raghu G, Rochwerg B, Zhang Y, Garcia CAC, Azuma A, Behr J, et al. An official ATS/ERS/JRS/ALAT clinical practice guideline: treatment of idiopathic pulmonary fibrosis. An update of the 2011 clinical practice guideline. Am J Respir Crit Care Med. 2015;192(2):e3-19. 10.1164/rccm.201506-1063ST. 26177183 10.1164/rccm.201506-1063ST

[3] Raghu G, Remy-Jardin M, Richeldi L, Thomson CC, Inoue Y, Johkoh T, et al. Idiopathic pulmonary fibrosis (an Update) and progressive pulmonary fibrosis in adults: an official ATS/ERS/JRS/ALAT clinical practice guideline. Am J Respir Crit Care Med. 2022;205(9):e18–47. 10.1164/rccm.202202-0399ST. 35486072 10.1164/rccm.202202-0399ST PMC9851481

[4] Podolanczuk AJ, Thomson CC, Remy-Jardin M, Richeldi L, Martinez FJ, Kolb M, et al. Idiopathic pulmonary fibrosis: state of the art for 2023. Eur Respir J. 2023;61(4):2200957. 10.1183/13993003.00957-2022. 10.1183/13993003.00957-2022 36702498

[5] Kaunisto J, Salomaa ER, Hodgson U, Kaarteenaho R, Kankaanranta H, Koli K, et al. Demographics and survival of patients with idiopathic pulmonary fibrosis in the FinnishIPF registry. ERJ Open Res. 2019;5(3):00170–2018. 10.1183/23120541.00170-2018. 31304177 10.1183/23120541.00170-2018 PMC6612605

[6] Raghu G, Chen S-Y, Yeh W-S, Maroni B, Li Q, Lee Y-C, et al. Idiopathic pulmonary fibrosis in US Medicare beneficiaries aged 65 years and older: incidence, prevalence, and survival, 2001–11. Lancet Respir Med. 2014;2(7):566–72. 10.1016/S2213-2600(14)70101-8. 24875841 10.1016/S2213-2600(14)70101-8

[7] King TE Jr, Bradford WZ, Castro-Bernardini S, Fagan EA, Glaspole I, Glassberg MK, et al. A phase 3 trial of pirfenidone in patients with idiopathic pulmonary fibrosis. N Engl J Med. 2014;370(22):2083–92. 10.1056/NEJMoa1402582. 24836312 10.1056/NEJMoa1402582

[8] Richeldi L, du Bois RM, Raghu G, Azuma A, Brown KK, Costabel U, et al. Efficacy and safety of nintedanib in idiopathic pulmonary fibrosis. N Engl J Med. 2014;370(22):2071–82. 10.1056/NEJMoa1402584. 24836310 10.1056/NEJMoa1402584

[9] Caminati A, Lonati C, Cassandro R, Elia D, Pelosi G, Torre O, et al. Comorbidities in idiopathic pulmonary fibrosis: an underestimated issue. Eur Respir Rev. 2019;28(153): 190044. 10.1183/16000617.0044-2019. 31578211 10.1183/16000617.0044-2019 PMC9488913

[10] Kreuter M, Ehlers-Tenenbaum S, Palmowski K, Bruhwyler J, Oltmanns U, Muley T, et al. Impact of comorbidities on mortality in patients with idiopathic pulmonary fibrosis. PLoS ONE. 2016;11(3): e0151425. 10.1371/journal.pone.0151425. 27023440 10.1371/journal.pone.0151425 PMC4811578

[11] George PM, Patterson CM, Reed AK, Thillai M. Lung transplantation for idiopathic pulmonary fibrosis. Lancet Respir Med. 2019;7(3):271–82. 10.1016/S2213-2600(18)30502-2. 30738856 10.1016/S2213-2600(18)30502-2

[12] Swigris JJ, Gould MK, Wilson SR. Health-related quality of life among patients with idiopathic pulmonary fibrosis. Chest. 2005;127(1):284–94. 10.1378/chest.127.1.284. 15653996 10.1378/chest.127.1.284

[13] Rautajoki T, Lehto JT, Sutinen E, Bergman P, Sintonen H, Rajala K, et al. Dyspnea associates with a widely impaired quality of life in idiopathic pulmonary fibrosis patients: a longitudinal study using 15D. J Palliat Med. 2023;26(10):1357–64. 10.1089/jpm.2022.0548. 37366772 10.1089/jpm.2022.0548

[14] Cox IA, Arriagada NB, De Graaff B, Corte TJ, Glaspole I, Lartey S, et al. Health-related quality of life of patients with idiopathic pulmonary fibrosis: a systematic review and meta-analysis. Eur Respir Rev. 2020;29(158):200154. 10.1183/16000617.0154-2020. 33153990 10.1183/16000617.0154-2020 PMC9488638

[15] Patel AS, Siegert RJ, Brignall K, Gordon P, Steer S, Desai SR, et al. The development and validation of the King’s Brief Interstitial Lung Disease (K-BILD) health status questionnaire. Thorax. 2012;67(9):804–10. 10.1136/thoraxjnl-2012-201581. 22555278 10.1136/thoraxjnl-2012-201581

[16] Kaunisto J, Kelloniemi K, Sutinen E, Hodgson U, Piilonen A, Kaarteenaho R, et al. Re-evaluation of diagnostic parameters is crucial for obtaining accurate data on idiopathic pulmonary fibrosis. BMC Pulm Med. 2015;15:92. 10.1186/s12890-015-0074-3. 26285574 10.1186/s12890-015-0074-3 PMC4541726

[17] Rajala K, Lehto JT, Sutinen E, Kautiainen H, Myllärniemi M, Saarto T. Marked deterioration in the quality of life of patients with idiopathic pulmonary fibrosis during the last two years of life. BMC Pulm Med. 2018;18(1):172. 10.1186/s12890-018-0738-x. 30458739 10.1186/s12890-018-0738-x PMC6247520

[18] Rajala K, Lehto JT, Sutinen E, Kautiainen H, Myllärniemi M, Saarto T. mMRC dyspnoea scale indicates impaired quality of life and increased pain in patients with idiopathic pulmonary fibrosis. ERJ Open Res. 2017;3(4):00084–2017. 10.1183/23120541.00084-2017. 29255720 10.1183/23120541.00084-2017 PMC5731772

[19] Seppälä S, Rajala K, Lehto JT, Sutinen E, Mäkitalo L, Kautiainen H, et al. Factor analysis identifies three separate symptom clusters in idiopathic pulmonary fibrosis. ERJ Open Res. 2020;6(4):00347–2020. 10.1183/23120541.00347-2020. 33043051 10.1183/23120541.00347-2020 PMC7533377

[20] Sinha A, Patel AS, Siegert RJ, Bajwah S, Maher TM, Renzoni EA, et al. The King’s Brief Interstitial Lung Disease (KBILD) questionnaire: an updated minimal clinically important difference. BMJ Open Respir Res. 2019;6(1): e000363. 10.1136/bmjresp-2018-000363. 30956799 10.1136/bmjresp-2018-000363 PMC6424243

[21] Mahler DA, Wells CK. Evaluation of clinical methods for rating dyspnea. Chest. 1988;93(3):580–6. 10.1378/chest.93.3.580. 3342669 10.1378/chest.93.3.580

[22] Bestall JC, Paul EA, Garrod R, Garnham R, Jones PW, Wedzicha JA. Usefulness of the Medical Research Council (MRC) dyspnoea scale as a measure of disability in patients with chronic obstructive pulmonary disease. Thorax. 1999;54(7):581–6. 10.1136/thx.54.7.581. 10377201 10.1136/thx.54.7.581 PMC1745516

[23] Phua G, Tan GP, Phua HP, Lim WY, Neo HY, Chai GT. Health-related quality of life in a multiracial Asian interstitial lung disease cohort. J Thorac Dis. 2022;14(12):4713–24. 10.21037/jtd-22-906. 36647495 10.21037/jtd-22-906 PMC9840018

[24] Kreuter M, Swigris J, Pittrow D, Geier S, Klotsche J, Prasse A, et al. Health related quality of life in patients with idiopathic pulmonary fibrosis in clinical practice: insights-IPF registry. Respir Res. 2017;18(1):139. 10.1186/s12931-017-0621-y. 28709421 10.1186/s12931-017-0621-y PMC5512739

[25] Tzouvelekis A, Karampitsakos T, Kourtidou S, Bouros E, Tzilas V, Katsaras M, et al. Impact of depression on patients with idiopathic pulmonary fibrosis. Front Med (Lausanne). 2020;7(7): 29. 10.3389/fmed.2020.00029. 32118014 10.3389/fmed.2020.00029 PMC7020231

[26] Prior TS, Hoyer N, Hilberg O, Shaker SB, Davidsen JR, Bendstrup E. Responsiveness and minimal clinically important difference of SGRQ-I and K-BILD in idiopathic pulmonary fibrosis. Respir Res. 2020;21(1):91. 10.1186/s12931-020-01359-3. 32316976 10.1186/s12931-020-01359-3 PMC7175493

[27] Wapenaar M, Patel AS, Birring SS, van Domburg RT, Bakker EWP, Vindigni V, et al. Translation and validation of the King’s Brief Interstitial Lung Disease (K-BILD) questionnaire in French, Italian, Swedish, and Dutch. Chron Respir Dis. 2017;14(2):140–50. 10.1177/1479972316674425. 28019103 10.1177/1479972316674425 PMC5720224

[28] Kim JW, Clark A, Birring SS, Atkins C, Whyte M, Wilson AM. Psychometric properties of patient reported outcome measures in idiopathic pulmonary fibrosis. Chron Respir Dis. 2021;18: 14799731211033925. 10.1177/14799731211033925. 34609156 10.1177/14799731211033925 PMC8495510

[29] Furukawa T, Taniguchi H, Ando M, Kondoh Y, Kataoka K, Nishiyama O, et al. The St. George’s respiratory questionnaire as a prognostic factor in IPF. Respir Res. 2017;18(1):18. 10.1186/s12931-017-0503-3. 28095852 10.1186/s12931-017-0503-3 PMC5240376

[30] Kreuter M, Swigris J, Pittrow D, Geier S, Klotsche J, Prasse A, et al. The clinical course of idiopathic pulmonary fibrosis and its association to quality of life over time: longitudinal data from the INSIGHTS-IPF registry. Respir Res. 2019;20(1):59. 10.1186/s12931-019-1020-3. 30876420 10.1186/s12931-019-1020-3 PMC6420774

[31] Glaspole IN, Chapman SA, Cooper WA, Ellis SJ, Goh NS, Hopkins PM, et al. Health-related quality of life in idiopathic pulmonary fibrosis: data from the Australian IPF Registry. Respirology. 2017;22(5):950–6. 10.1111/resp.12989. 28166611 10.1111/resp.12989

[32] Birring SS, Bushnell DM, Baldwin M, Mueller H, Male N, Rohr KB, et al. The psychometric properties of the King’s Brief Interstitial Lung Disease questionnaire and thresholds for meaningful treatment response in patients with progressive fibrosing interstitial lung diseases. Eur Respir J. 2022;59(6):2101790. 10.1183/13993003.01790-2021. 34764181 10.1183/13993003.01790-2021 PMC9160394

