# Supplementary material for: Health-related quality of life measured with K-BILD is associated with survival in patients with idiopathic pulmonary fibrosis
Source: BMC Pulm Med. 2024 Sep 30;24:480. doi: 10.1186/s12890-024-03303-3 (PMC11443770; doi:10.1186/s12890-024-03303-3)
Supplement: Supplementary file 1 — Supplementary Material 1. [file 12890_2024_3303_MOESM1_ESM.pdf]

Additional file 1.

K-BILD translation procedure

1. Approval of the developer of the K-BILD questionnaire (Q1) for the translation process.
2. Translation of the K-BILD from English into Finnish (Q1)(→T1).
3. The Finnish translation (T1) was evaluated and approved by a committee of Finnish physicians (→T2).
4. Back-translation of the Finnish translation (T2) into English by a qualified translator (→Q2).
5. A quality-of-life specialist reviewed and ensured the contents of the back-translated document (Q2) (→T3).
6. Five patients with interstitial lung disease completed the Finnish translation (T3) and were interviewed (→T4).
7. The Finnish K-BILD (T4) was back-translated into English by a qualified translator (→Q3).
8. Approval of the back-translated K-BILD (Q3) by the developer of the original K-BILD.
