# Supplementary material for: Health-related quality of life measured with K-BILD is associated with survival in patients with idiopathic pulmonary fibrosis
Source: BMC Pulm Med. 2024 Sep 30;24:480. doi: 10.1186/s12890-024-03303-3 (PMC11443770; doi:10.1186/s12890-024-03303-3)
Supplement: Supplementary file 2 — Supplementary Material 2. [file 12890_2024_3303_MOESM2_ESM.pdf]

Additional file 2.

Pairwise log rank comparisons were conducted to determine which intervention groups had different survival distributions. Bonferroni correction was performed with a statistical significance accepted at the $p < 0.008$ level. There was a statistically significant difference in survival distributions for Group A vs. Group B ($\chi^2(1) = 9.115$, $p = 0.003$), Group A vs. Group C ($\chi^2(1) = 26.080$, $p < 0.001$), and Group A vs. Group D ($\chi^2(1) = 27.800$, $p < 0.001$). However, the survival distributions for Group B and Group C ($\chi^2(1) = 4.539$, $p = 0.033$), Group B and Group D ($\chi^2(1) = 5.685$, $p = 0.017$), and Group C and Group D ($\chi^2(1) = 0.186$, $p = 0.667$) were not statistically significantly different.
