# Supplementary material for: Health-related quality of life measured with K-BILD is associated with survival in patients with idiopathic pulmonary fibrosis
Source: BMC Pulm Med. 2024 Sep 30;24:480. doi: 10.1186/s12890-024-03303-3 (PMC11443770; doi:10.1186/s12890-024-03303-3)
Supplement: Supplementary file 3 — Supplementary Material 3. [file 12890_2024_3303_MOESM3_ESM.pdf]

Additional file 3.

Figure. ROC curve for the K-BILD total score to predict a survival time of less than two years.

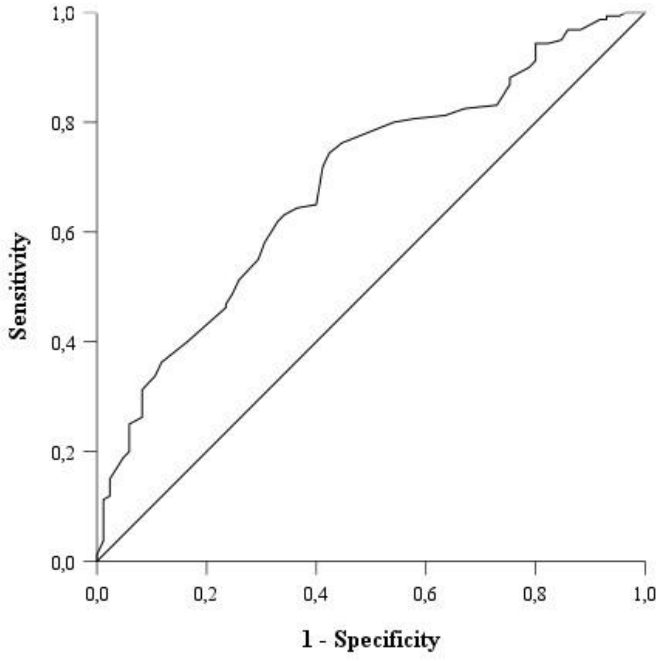

ROC, receiver operating characteristic; K-BILD, King's Brief Interstitial Lung Disease Questionnaire.
