# Supplementary material for: Health-related quality of life measured with K-BILD is associated with survival in patients with idiopathic pulmonary fibrosis
Source: BMC Pulm Med. 2024 Sep 30;24:480. doi: 10.1186/s12890-024-03303-3 (PMC11443770; doi:10.1186/s12890-024-03303-3)
Supplement: Supplementary file 4 — Supplementary Material 4. [file 12890_2024_3303_MOESM4_ESM.pdf]

Additional file 4.

Table 3. Patient characteristics according to the survival (short- and long-term survivors).

	Survived < 2 years	Survived > 2 years	P-value
**Total**, n (%)	85 (34.7)	160 (65.3)	
**Gender**, n (%)			0.174
Females	24 (28.2)	59 (36.9)	
Males	61 (71.8)	101 (63.1)	
**Age**, Mean (SD), years	75.1 (8.2)	73.9 (9.0)	0.431
**Smoking status**, n (%)			0.034
Never	32 (37.6)	84 (52.5)	
Former	46 (54.1)	59 (36.9)	
Current smoker	7 (8.2)	17 (10.6)	
**Comorbidities**, n (%)			
Hypertension	35 (41.2)	66 (41.3)	0.991
Coronary artery disease	20 (23.5)	34 (21.3)	0.682
Diabetes mellitus	22 (25.9)	26 (16.3)	0.071
COPD	17 (20.0)	26 (16.3)	0.463
Asthma	6 (7.1)	17 (10.6)	0.362
Cancer	13 (15.3)	28 (17.5)	0.660
**Lung function**			
FVC Median (IQR) % of predicted	76.0 (67.0-91.0)	84.5 (73.3-95.0)	0.007
DLCO Median (IQR) % of predicted	54.0 (46.0-63.3)	60.0 (52.3-74.0)	< 0.001

COPD, chronic obstructive pulmonary disease; FVC, forced vital capacity; DLCO, diffusing capacity of the lungs for carbon monoxide.
