# Supplementary material for: Health-related quality of life measured with K-BILD is associated with survival in patients with idiopathic pulmonary fibrosis
Source: BMC Pulm Med. 2024 Sep 30;24:480. doi: 10.1186/s12890-024-03303-3 (PMC11443770; doi:10.1186/s12890-024-03303-3)
Supplement: Supplementary file 5 — Supplementary Material 5. [file 12890_2024_3303_MOESM5_ESM.pdf]

Additional file 5.

Post hoc analysis revealed statistically significant differences in K-BILD total scores between the following time points:Category of mMRC 0-1: 0 to 24 months ($p < 0.001$), 0 to 30 months ($p < 0.001$), 6 to 12 months ($p = 0.009$), 6 to 18 months ($p = 0.023$), 6 to 24 months ($p < 0.001$), and 6 to 30 months ($p < 0.001$). Category of mMRC 2-4: 0 to 30 months ($p = 0.04$), 6 to 12 months ($p = 0.018$), 6 to 24 months ($p = 0.023$), and 6 to 30 months ($p = 0.006$).
